# Heterogenization of Ketone Catalyst for Epoxidation by Low Pressure Plasma Fluorination of Silica Gel Supports

**DOI:** 10.3390/molecules22122099

**Published:** 2017-11-30

**Authors:** Lucia D’Accolti, Nicoletta De Vietro, Fiorenza Fanelli, Caterina Fusco, Angelo Nacci, Francesco Fracassi

**Affiliations:** 1 Department of Chemistry, University of Bari “Aldo Moro”, Via Orabona 4, 70126 Bari, Italy; nicoletta.devietro@uniba.it (N.D.V.); angelo.nacci@uniba.it (A.N.); 2 ICCOM-CNR, SS Bari, Via Orabona 4, 70126 Bari, Italy; fusco@ba.iccom.cnr.it; 3 NANOTEC-CNR, c/o Department of Chemistry, University of Bari “Aldo Moro”, Via Orabona 4, 70126 Bari, Italy; Fiorenza.fanellinr@cnr.it

**Keywords:** dioxiranes, PE-CVD, epoxidation, fluorous chemistry

## Abstract

Low pressure plasma was used for preparing heterogeneous organocatalysts **2**-(**A**)-(**C**) suitable for dioxirane-mediated epoxidations. Heterogenization was accomplished by adsorption of the methyl perfluoroheptyl ketone (**2**) on fluorinated supports (**A**)-(**C**) deriving from the treatment of commercial C_8_-silica gel in low pressure plasma fed with fluorocarbons. Catalyst **2**-(**C**) proved to be the most efficient one, promoting epoxidation of an array of alkenes, including unsaturated fatty esters like methyl oleate (**10**) and the triglyceride soybean oil (**11**), with the cheap potassium peroxymonosulfate KHSO_5_ (caroate) as a green oxidant. Notably, the perfluorinated matrix gives rise to the activation of caroate, generating singlet oxygen. Materials were characterized by infrared Attenuated Total Reflectance spectroscopy (ATR-FTIR), X-ray Photoelectron Spectroscopy (XPS ) and Emission Scanning Electron Microscope (FESEM).

## 1. Introduction

Fluorous chemistry is a well-known methodology that involves the use of perfluorinated solvents and/or perfluorinated reagents to facilitate catalyst or reaction products recovery [1]. Typically, conventional organic reagents incorporating perfluorinated tags can react in fluorous solvents (e.g., perfluorohexane) and are then extracted into organic phases, or can be supported on fluorous solid matrixes behaving as heterogeneous reactants or catalysts [1,2].

Well-known benefits of this strategy can be found into the increased reaction rates of alkene epoxidation with H_2_O_2_ performed into perfluoroalcohols (e.g., 2,2,2-trifluoroethanol or 1,1,1,3,3,3-hexafluoro-2-propanol, HFIP) [2], or with perfluorinated ketones/caroate [3]. However, the use of perfluorinated solvent suffers from the limitation of the prohibitive costs for large-scale applications.

To overcome this drawback, Fluorous Silica Gel (FSG) systems, that can be used directly as a catalyst [4], or as solid supports to heterogenize perfluorinated catalytic species by means of fluorous interactions [5,6] are most useful. Representative examples are given by perfluoro-tagged gold nanoparticles immobilized on FSG as suitable catalysts for oxidation of alkenes with H_2_O_2_ [7], or immobilized fluorinated flavins useful for promoting the photocatalytic oxidation of benzyl alcohols [8].

**Scheme 1:**
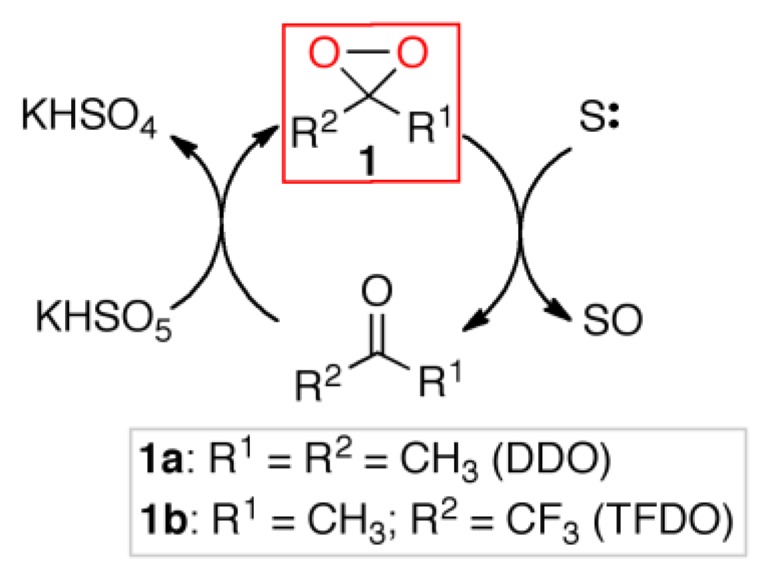
Generation of dioxirane in situ from ketone/caroate.

In this context, the oxidations mediated by organo-catalyst dioxiranes **1** can find special applications [9,10], alternative to metal catalysis [11,12]. The dioxiranes are very active and selective oxidants, the most used being dimethyldioxirane (DDO) and methyl(trifluoromethyl)dioxirane (TFDO), which can be used either as isolated stoichiometric reagents [13] or as oxidizing organocatalysts, generated in situ by reacting parent ketones with cheap oxidants such as potassium caroate [14] (Scheme 1).

In the last decade, anchoring of the trifluoroacetyl moiety of dioxiranes by grafting onto supports like poly(ethylene) glycol (PEG) or silica gel furnished very active epoxidizing systems of alkenes [15,16]. In this context, we recently reported the first application of plasma-enhanced chemical vapor deposition (PE-CVD) to the synthesis of supported perfluorinated ketones, by covering Merrifield resin beads with an oxygen-containing fluorocarbon thin film deposited in a hexafluoropropene-O_2_ low pressure non equilibrium plasma [17]. This surface modification approach has to be considered particularly valuable from environmental standpoint since no solvents are required and no dangerous wastes are produced [18,19].

The major advantage of this versatile technique resides in the fact that plasma treatment usually changes the characteristics of the upper layers of the treated material surface without damaging its bulk properties, thanks to the very low range of penetration.

Interestingly, catalytic activity of supported materials was found to be dependent on the plasma deposition conditions, leading either to the selective epoxidation of alkenes (mediated by dioxirane) or preferentially to their oxidative cleavage, depending on whether C_3_F_6_-O_2_ or pure C_3_F_6_ were used to feed the plasma.

Given the success of the original method [17], we decided to extend these findings developing a new and more simple approach for dioxirane heterogenization, by anchoring its perfluorinated parent ketone to new fluorous supports obtained obtained by depositing a perfluoropolymer from perfluoropropene C_3_F_6_, analogous to commercial FSG, by simple fluorous interactions. 

The use of low pressure non equilibrium plasmas assured the fine preparation of the fluorinated matrix suitable for a prompt anchoring of dioxirane precursor, which was chosen to be methyl perfluoroheptyl ketone C_7_F_15_COCH_3_ (**2**), a fluorinated ketone previously described as an analogous of trifluoroacetone into the in situ generation of dioxirane [20].

Anchoring of methyl perfluoroheptyl ketone (**2**) avoids the drawback of its large scale utilization, due to its high cost, and overcomes the problem of its low solubility in MeCN/water, which imposes the use of the expensive solvent HFIP [20]. In addition, this approach would permit the easy preparation and handling of the catalyst, a major efficiency in caroate mediated oxidation without leaching, and a large scope of olefinic substrates, including fatty acid esters like methyl oleate and soybean oil. Herein, the synthesis and catalytic activity of this plasma prepared heterogenized organocatalyst is disclosed.

## 2. Results and Discussion

Initially, the catalyst supports were prepared by treating commercial C_8_-silica gel in low pressure plasmas fed with tetrafluoromethane (CF_4_) or hexafluoropropene (C_3_F_6_). The latter reactant typically ensures the deposition of thin films with high F-to-C ratios [21]. Tetrafluoromethane, on the other hand, is generally employed as an etchant gas or for the surface grafting of fluorinated moieties rather than to deposit a fluorocarbon thin films [21].

**Figure 1:**
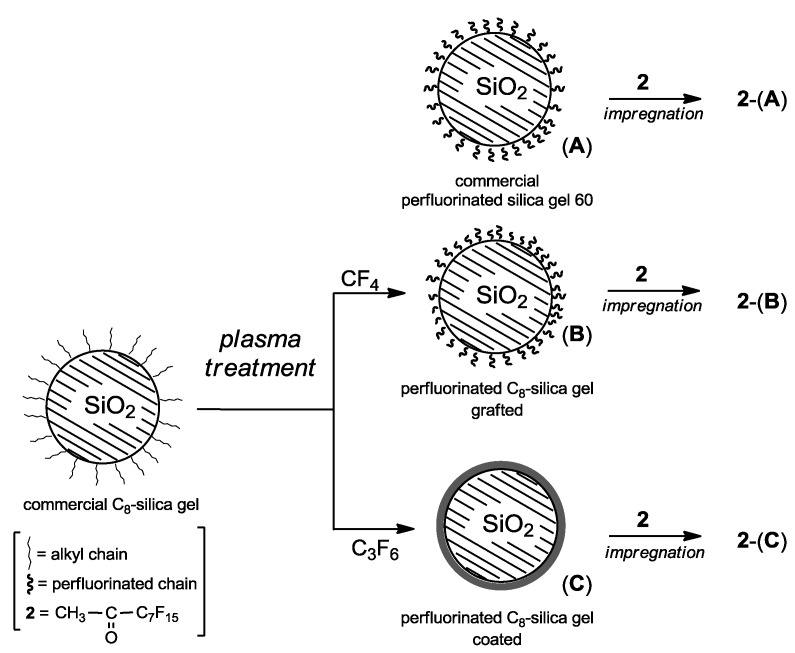
Preparation of heterogeneous perfluorinated organocatalysts.

Applying these protocols, commercial C_8_-silica gel was modified leading to the two different perfluorinated supports: (**B**) bearing grafted perfluorinated chains, and (**C**) coated with a perfluorinated thin film (Figure 1). For comparison, the commercial perfluorinated silica gel 60 without plasma treatment (**A**) was also used as support. Afterwards, impregnation of silica gel supports (**A**)-(**C**) with methyl perfluoroheptyl ketone (**2**), afforded the corresponding organocatalyst composites **2**-(**A**), **2**-(**B**) and **2**-(**C**) to be used as dioxirane precursors in alkene epoxidation.

Catalytic performances of these materials were evaluated in the model epoxidation reaction of *trans*-β-methylstyrene (**3**) with potassium caroate (KHSO_5_) and compared with those of perfluoroketone (**2**) used alone under homogeneous conditions (Table 1, run 1).

Reaction parameters were calibrated for processing 1 mmol of alkene in the presence of ketone (**2**) (in the range of 5–15%) and caroate (4 mmol) as an oxygen source. Reaction medium and the other settings were chosen to be quite similar to those generally adopted in the dioxirane epoxidation in situ; in particular, the conditions used in the case of silica functionalized with trifluoroacetyl moiety [16]. Despite the heterogeneous conditions, supported organocatalysts proved to be highly active, providing almost all higher conversions in shorter reaction times respect to those obtained with methyl perfluoroheptyl ketone (**2**) alone (Table 1, runs 3–4). As expected for a heterogeneous process, selectivities were slightly lowered, due to the formation of benzaldehyde (**3b**) as a byproduct resulting from the complete cleavage of the double bond. In addition, catalysts **2**-(**A**) and **2**-(**B**) suffered from the disadvantage of leaching of the dioxirane precursor **2** from the solid surface during the reaction (Table 1, runs 2–3).

In contrast, catalyst **2**-(**C**) showed higher performance, giving the highest conversion and selectivity without apparent desorption of methyl perfluoroheptyl ketone (**2**) (no leaching, Table 1, run 4). Therefore, **2**-(**C**) was used in the successive experiments, where the influence of the catalyst loading was investigated by using variable amounts of (**2**) adsorbed on support (Table 1, runs 5–7).

From the screening clearly emerged that the increase of that adsorbed precursor up to 15% ca. did not result in an increment of conversion and selectivity, but solely in a reduced reaction time (Table 1, run 5). In contrast, the decrease of (**2**) down to 5% ca. led to the formation of much higher amounts of the oxidative cleavage compound benzaldehyde (**3b**) (Table 1, run 7). In line with this trend, when reaction was performed in the presence of the sole perfluorinated support (**C**), (**3b**) was the sole product observed (Table 1, run 8).

**Figure 2:**
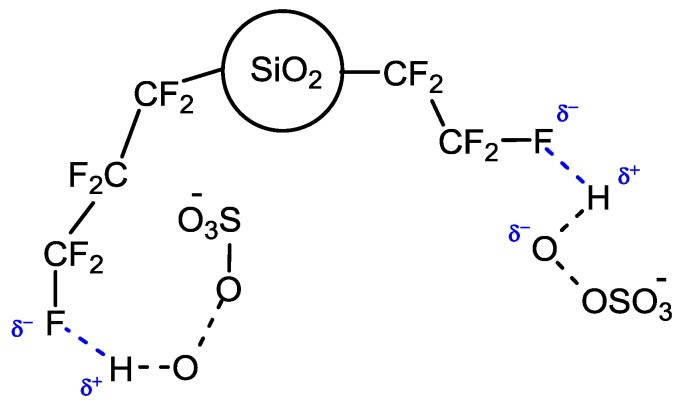
Proposed mechanism for caroate activation by fluorinated silica gel support.

The rationale for this switching of selectivity is the probable activating effect exerted by fluorous silica gel (prepared by plasma processing) towards the oxygen source caroate. The activating mechanism, not yet completely understood, assumes that perfluoroalkyl chains on the support surface could share the fluorine atoms affording a multiple weak hydrogen-bond network [4], thus establishing a strong interaction with caroate that should lead to a more facile O–O peroxide bond cleavage (Figure 2). 

The activated KHSO_5_ should immediately react with adsorbed ketone (**2**), leading to epoxidation via dioxirane (**1**) (Scheme 1). Otherwise, in the absence of the methyl perfluoroheptyl ketone, it should undergo the self-decomposition reaction according to Equation (1), thus releasing single oxygen ^1^O_2_ which is probably responsible for the oxidative cleavage of the double bond to give benzaldehyde [3,22,23].

(1) HSO5−+SO5→HSO4−+SO42−+O21,

This latter hypothesis was confirmed by the reaction outcome of epoxidation carried out in the presence of the singlet oxygen quencher NaN_3_, for which selectivity turned to be completely in favor of the epoxide product (**3a**) (Table 1, run 9) [24,25]. Note to mention, the activating effect of the perfluorinated matrix allowed to achieve a double advantage: (i) a higher reactivity of heterogeneous catalyst **2**-(**C**) respect to methyl perfluoroheptyl ketone (**2**) and (ii) a major flexibility of the protocol, enabling the possibility of performing two different kinds of olefin transformations (epoxidation or double bond cleavage) by means of the simple choice of starting reagents.

**Figure 3:**
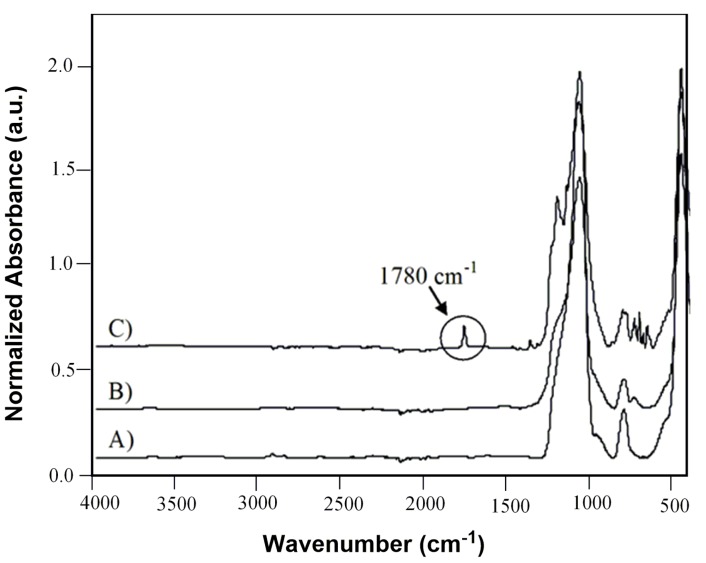
ATR-FTIR spectra of C_8_ Silica gel (**A**) pristine, (**B**) after plasma coating and (**C**) after plasma coating and ketone (**2**) impregnation.

After the optimization experiments, the novel heterogeneous perfluorinated silica gel plasma coated catalyst **2**-(**C**) was fully characterized by ATR-FTIR, XPS and SEM techniques. Infrared spectra in Figure 3 clearly confirmed the adsorption of (**2**) on the surface of plasma treated support (**C**) evidenced by the carbonyl band, centered at 1780 cm^−1^ ν (C=O), which appeared into the spectrum of coated silica gel after impregnation [26,27].

The XPS atomic composition of the supports surface (Table 2) clearly evidenced the high degree of fluorination obtained in the case of the coated support (**C**), as indicated by the high F concentration (Table 2, run 4). This should explain the superior catalytic performance of the impregnated catalyst **2**-(**C**) together with its better capacity in retaining adsorbed methyl perfluoroheptyl ketone (**2**) without leaching. It is worth mentioning that the XPS data also evidenced that during impregnation, a partial detachment of the fluorinated coating occurs, as demonstrated by the increase of atomic concentration of the underlying silicon from 1% to 7% (Table 2, runs 4–5, see also Suppo Info Table S1, Figures S1 and S2).

**Figure 4:**
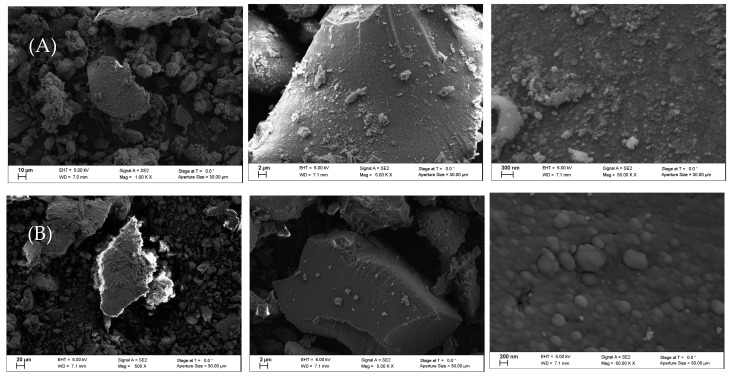
(**A**) Pristine silica powder; (**B**) silica powder with perfluoro plasma polymer.

Finally, the scanning electron microscopy (SEM) images reported in Figure 4 show that the silica surface was coated by the perfluoro polymers (Figure 4B).

**Figure 5:**
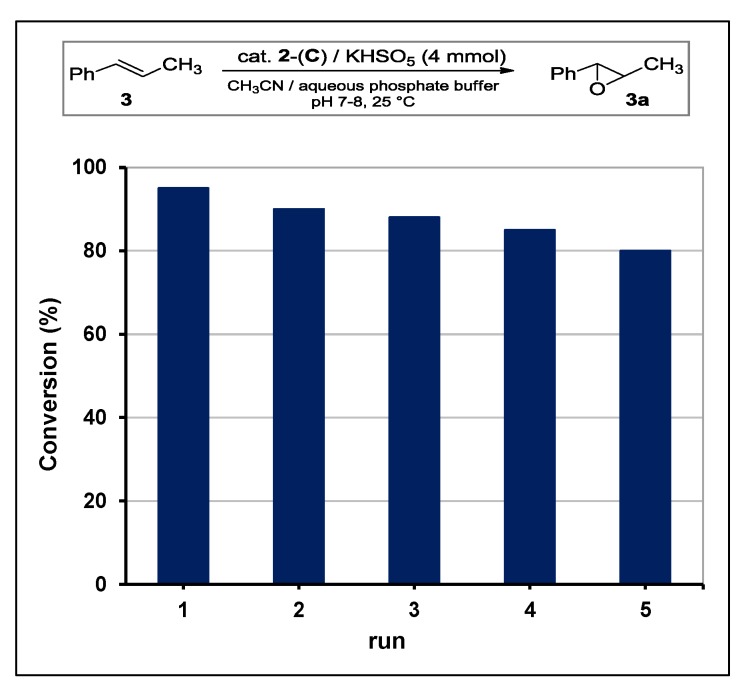
Recycling of **2**-(**C**) organocatalysts in the epoxidation of alkene **3** with caroate.

The stability of catalyst **2**-(**C**) was studied in a series of recycling experiments (Figure 5). Being the ketone (**2**) solely adsorbed on support surface by means of fluorous interactions, recycling procedures were accomplished in a careful manner to avoid desorption. After each run, the solid residue was decanted and supernatant aqueous buffer was removed with care washing with little aliquots of fresh solvent. Then, fresh buffer and reagents were added to the solid for a new run (see Section 3.4). As shown in Figure 5, catalytic performances for the first five runs remained above 80% of conversion.

The evident reduction of catalytic activity was due to both the partial desorption of methyl perfluoroheptyl ketone (detected by GLC analyses of mother liquor) and the accumulation of potassium sulfate by-product.

However, to evaluate the whole catalyst robustness, epoxidation of *trans*-β-methyl styrene (**3**) was carried out with excess amounts of alkene and monitored until catalyst deactivation (see Section 3.5). Under these conditions a total turnover number (TON) of 206 ca. was observed, indicating that the catalyst is stable and much more active respect to its homogeneous counterpart [17] (see also Table 1, run 1).

**Figure 6:**
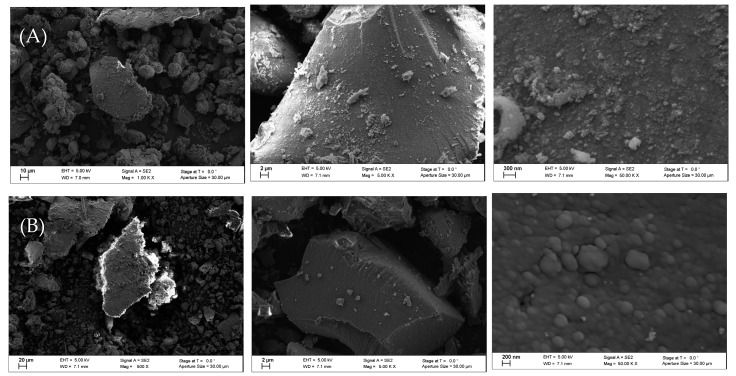
(**A**) Exhausted catalyst **2-**(**C**); (**B**) regenerated catalyst **2-**(**C**)**.**

The loss of catalytic activity of **2**-(**C**) was explained with the partial removal of plasma fluoropolymer from the surface of silica support as a consequence of reaction conditions; most probably due to the mechanical damage of stirring (Figure 6A). This assumption was verified re-submitting deactivated catalyst to C_3_F_6_–O_2_ plasma treatment that restored the perfluoropolymer coating and consequently, after re-impregnation of methyl perfluoroheptyl ketone (**2**), the catalyst performance, as demonstrated by the high conversion (95%) and selectivity (90%) displayed by the regenerated **2**-(**C**) in the epoxidation of (**3**).

The substrate scope of **2**-(**C**) was studied with an array of olefin substrates (Table 3). Epoxidation of cyclohexene (**4**) took place with 98% of conversion after 2 h and the cyclohexene oxide (**4a**) product was obtained with excellent selectivity (Table 3, run 1). Similarly, the allylic alcohol cyclohex-2-enol (**5**) reached full conversion, affording the corresponding epoxide (**5a**) together with a 15% of cyclohex-2-enone (**5b**) [28] (Table 3, run 2). In contrast, 1-octene (**6**) afforded only a 15% of conversion (Table 3, run 3), while no epoxidation occurred with the more deactivated substrate *trans*-cinnamonitrile (**7**), which rather partially underwent the double bond cleavage affording benzoic acid (Table 3, run 4).

Extending the substrate scope to the selective oxidation of key organic compounds, the protocol was also applied to a representative substrate of each of the following class of compounds: an alkyne (3-hexyne (**8**)), an alcohol (3-hexanol (**9**)), an unsaturated natural monoester (methyl oleate (**10**)) and an unsaturated triglyceride (soybean oil (**11**)).

Oxidation of 3-hexyne (**8**) afforded smoothly the expected 3,4 hexanedione (**8a**) (Table 1, run 5), a very interesting result if considering that 1,2-diones are valuable building blocks in organic synthesis, especially as precursors of several heterocyclic compounds [29,30,31]. This representative example shows how the novel catalyst is more reactive than those reported previously [16,20], including also the methyl perfluoroheptyl ketone **2** used under homogeneous conditions [20].

In contrast, 3-hexanol (**9**) afforded only trace amounts of expected ketone (**9a**), even after using excess amounts of caroate and prolonged reaction times (48 h). This indicates that this protocol is highly selective towards epoxidation and can tolerate other functional groups susceptible to oxidation.

Finally, epoxidation of fatty acid esters (**10**)and (**11**) (Table 3, runs 7–8) [32] further validates the efficacy of this method that can be used even in the production of epoxidized soybean oil (ESO) (**11a**), a bulk chemical of great practical importance largely used as plasticizer, lubricant, cross-linking agent, stabilizer etc. [33].

## 3. Experimental Section

### 3.1. General Information

The gas chromatography (GLC) analyses were performed using a GC-2010 Plus (SHIMADZU Duisburg, Germany) gas chromatograph equipped with a flame ionization detector (FID) and a ZB-1 column (100% dimethylpolysiloxane stationary phase, 30 m × 0.2 µm ID) utilizing n-butanol as the internal standard for calibration. The GC-MS (gas chromatography-mass spectrometry) analyses were run on a GCMS QP2010 SE (SHIMADZU Duisburg, Germany) using a ZB-1 column (30 m × 0.25 µm id) with the MS detector in electron impact (EI) mode (70 eV).

A vacuum Fourier transform infrared spectrometer Bruker Vertex 70v Fourier transform infrared (Buker, Milan, Italy). was used to record the infrared absorption spectra (400–4000 cm^−1^ range, 4 cm^−1^ resolution) of all catalytic supports. The spectra were acquired utilizing an ATR module equipped with a single reflection diamond ATR crystal (refractive index of 2.4). Each analysis was repeated on three samples. 

The surface chemical analyses of the pristine, plasma-coated and used supports were carried out by means of XPS using a using a Theta probe spectrometer (Thermo Electron Corporation, Waltham, MA, USA) spectrometer equipped with a monochromatic Al KaX-ray source (1486.6 eV), operating at a spot size of 300 µm corresponding to a power of 70 W. Survey (0–1200 eV) and high resolution (C1s, O1s, F1s and Si2p) spectra were recorded in FAT (fixed analyzer transmission) mode at pass energy of 200 and 100 eV, respectively. All spectra were acquired at a take-off angle of 37° with respect to the sample surface. A flood gun was used to balance the surface charging. The C1s signal for the hydrocarbon component (285.0 eV) was used as internal standard for charging correction. For the quantitative analysis, repeated measurements allowed to register a maximum relative standard deviation of about 3%. Atomic percentages were calculated from the high resolution spectra using the Scofield sensitivity factors set in the XPS data processing software and a non-linear Shirley background subtraction algorithm.

The morphology of the silica powders was investigated by a SUPRA 40 field emission scanning electron microscope (FESEM, Zeiss ITALIA, Milan, Italy). SEM images were acquired with a Everhart–Thornley detector at theworking distance in the range 7–7.5 mm, electron acceleration voltage (extra-high tension, EHT) of 5 kV, magnification in the range 0.1–50 KV. The silica powders were fixed on ultrahigh vacuum compatible carbon tape and sputter-coated with a 20 nm thick layer of Cr prior to SEM observation utilizing a turbo-pumped sputter coater (model Q150T, Quorum Technologies, Laughton, East Sussex, UK)

Commercial solvents and reagents of high purity were employed. Commercial (Aldrich, Milan, Italy) silica gel C_8_ and perfluorinated silica gel 60 were 200–450 mesh. Co-solvent water was freshly doubly distilled before use. Commercial (Degussa, Essen, Germany) Caroat^®^ triple salt (2 KHSO_5_·KHSO_4_·K_2_SO_4_, iodometry 2.86 mmol/g) was our source of potassium monoperoxysulfate. The soybean oil (iodine number of 130 g I_2_/100 g) was provided by Valsoia (Bologna, Italy).

### 3.2. Catalyst Preparation

#### 3.2.1. PE-CVD Treatment of C_8_-Silica Gel Support

**Figure 7:**
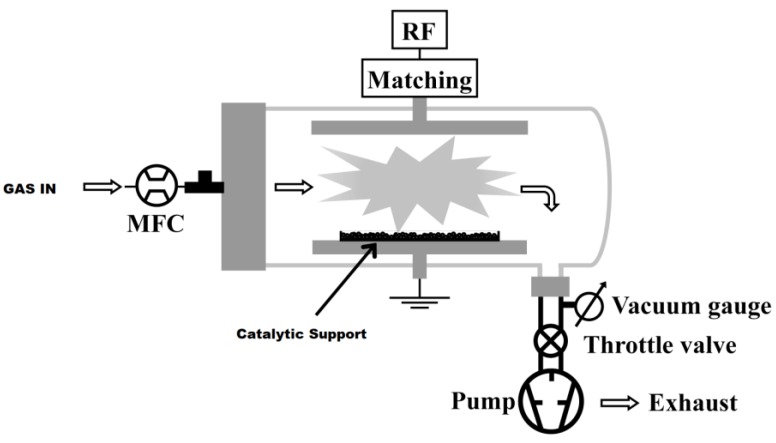
Schematic of the low pressure plasma reactor utilized for the silica gel treatment.

Commercial powder C_8_-silica gel was treated in the parallel plate, low pressure plasma reactor shown in Figure 7.

The reactor was composed of a 68 cm long Pyrex glass tube (9 cm internal diameter) closed on one side by a metallic flange sealed with Viton o-rings and containing two rectangular stainless steel electrodes (19 × 7 cm^2^). The lower electrode was grounded, while the upper one was connected to a radio frequency (RF, 13.56 MHz) power supply by means of an L-type matching network. The feed gas flow rate was controlled by electronic mass flow controllers (MFC), and the pressure was measured by a capacitance vacuum gauge. A rotative pump and a manual throttle valve allowed for a constant working pressure during the surface modification processes. During the PE-CVD experiments, the plasma was fed with hexafluoropropene (C_3_F_6_) at a flow rate of 30 sccm, and the input power was fixed at 50 W. The grafting processes was performed with CF_4_ at a flow rate of 20 sccm and 20 W input power. Plasma treatments were carried out on 1.0 g of catalytic support distributed over a flat stainless steel container positioned on the grounded electrode.

#### 3.2.2. Preparation of Supported Catalysts by Impregnation

The composite materials were prepared by impregnation of C_8_ silica gel supports **A**-**C** with an appropriate quantity of methyl perfluoroheptyl ketone (**2**). Typically, ketone (**2**) was dissolved in 2.5 mL of perfluorohexane and 1 g of finely ground silica support was added to this solution. The resulting dispersion was stirred overnight at room temperature and afterwards the solid was separated by centrifugation and dried. The GC/MS analysis of the supernatant solution shows that ketone (**2**) is completely absorbed. Silica gel composites thus obtained, labelled as **2**-(**A**), **2**-(**B**) and **2**-(**C**), were typically loaded with of ketone (**2**) in the range of 5 × 10^−2^–15 × 10^−2^ mmol per 100 mg of support.

### 3.3. Epoxidation Procedure

The oxidation of substrates was carried out in a batch reactor operating at a fixed temperature (25 °C). In a typical experiment, the substrate (**3**–**9**, 1 mmol) was added to a suspension of catalyst **2**-(**C**) (100 mg) in CH_3_CN (5 mL) and aqueous phosphate buffer (5 mL, 0.8 M, pH 7.5), also containing Na_2_EDTA (4.0 g/L ca.) to sequester any traces of heavy metals. Under vigorous stirring, solid caroate (1.32 g, 4 mmol) was added portionwise over 10–30 min, and the resulting mixture was allowed to react at 20–25 °C for an appropriate amount of time (1–12 h). The reaction progress was monitored as follows: aliquots (0.2 mL), periodically withdrawn from the reaction flask, were quickly filtered through a cotton plug to remove the solid catalyst, diluted to 2 mL with acetonitrile containing the internal standard, and analysed by GC. In each experiment, the values were measured at various reaction times; data from two or more independent runs were averaged (estimated error is ±3%). Upon reaction completion, the reaction mixture was partitioned between water (10 mL) and CH_2_Cl_2_ (10 mL) then filtered through a Buchner funnel to remove the suspended catalyst. The organic layer was separated and dried over MgSO_4_, and the solvent was removed under reduced pressure to afford the crude products identified by GC-MS analysis. In most of the cases, pure epoxide products (95–97%, GC) were obtained by flash chromatography (silica gel, *n*-hexane/Et_2_O). The isolated yields were determined by considering the total amount of substrate, usually 1 mmol. The products were characterized by ^1^H-NMR, GC-MS, and identified by comparison with this data with the spectral data of authentic samples.

*(E)-β-Methylstyrene oxide* (**3a**) [34,35]. (107 mg (0.80 mmol), 80% isolated yield, GC purity 95%): colorless oil, bp 93–96 °C/16 mmHg.

*Cyclohexene oxide* (**4a**) [35,36]. (92 mg (0.94 mmol, 94% isolated yield GC purity 95%) colorless oil, bp 129–130 °C.

*3-Hydroxycyclohex-1-ene oxide* (**5a**) [28]. (87 mg (0.76 mmol), 76% isolated yield, GC purity 95%): colorless oil, *syn*/*anti* ratio 60/40 (GC).

*Cyclohex-2-enone* (**5b**) [28]. (12 mg (0.13 mmol), 13% isolated yield, GC purity 95%).

*1-Octene oxide* (**6a**) [35]. (16 mg (0.12 mmol), 13% isolated yield, GC purity 97%; recovered 1-octene (**6**) substrate: 94 mg (0.84 mmol)): colorless oil, bp 62–65 °C/17 mmHg.

*Hexane-3,4-dione* (**8a**) [29]. (108 mg (0.95 mmol), 95% isolated yield, GC purity 95%): colorless oil, 131–133 °C.

*Methyl oleate oxide* (**10a**) [32,37]. (260 mg, 83% isolated yield, GC purity 90%): colorles oil.

*Epoxidized soybean oil (ESO)* (**11a**) [32,38]. see ^1^H-NMR in Supplementary Materials.

### 3.4. Recycling Experiments

The stability of catalyst **2**-(**C**) was studied in a series of recycling experiments. In the first run, substrate **3** (1 mmol) was added to a suspension of 100 mg of catalyst **2**-(**C**) in CH_3_CN (5 mL) and aqueous phosphate buffer (5 mL, 0.8 M, pH 7.5), also containing Na_2_EDTA (4.0 g/L ca.) to sequester any trace of heavy metals. Under vigorous stirring, solid caroate (1.32 g, 4 mmol) was added portionwise over 10–30 min, and the resulting mixture was allowed to react at 20–25 °C for 4 h.

After completion of reaction, the solid residue was decanted and supernatant aqueous buffer was carefully removed washing with little aliquots of fresh solvent. Then, fresh buffer and reagents were added to the solid for a new run.

After each cycle, aqueous supernatant was treated for evaluating conversions by partition between water (10 mL) and CH_2_Cl_2_ (10 mL). The organic layer was separated and dried over MgSO_4_, and the solvent removed under reduced pressure to afford the crude products identified by GC-MS analysis.

### 3.5. TON Measurement

To evaluate the whole catalyst robustness, epoxidation of *trans*-β-methyl styrene (**3**) was carried out with excess amounts of alkene and monitored until deactivation. In a typical experiment, to 2.50 g (21.186 mmol) of substrate (**3**) and 0.100 g (bearing adsorbed 9.7 × 10^−2^ mmol of ketone (**2**) of catalyst **2**-(**C**) in acetonitrile (5 mL) and aqueous buffer (10 mL, pH 8), caroate (1.40 g, 4 mmol) was added portion wise together with further buffer aliquots (5 mL) during 48 h. After addition of a total amount of 33.0 g (95 mmol) of caroate, no further progress in alkene conversion (95%, ca. 48 h) was detected (GC monitoring). Based on the substrate amount converted (20 mmol) and the catalyst loading (0.097 mmol) the calculated turnover number (TON) value was 206.

## 4. Conclusions

In summary, a novel heterogeneous catalyst for epoxidation reactions was prepared by the modification of the surface of commercial C_8_-silica gel with non-equilibrium low pressure plasma fed with hexafluoropropene (C_3_F_6_). The successive impregnation with ketone (**2**) of the C_8_-silica gel coated with a thin perfluorinated film, afforded the organocatalyst **2**-(**C**) suitable for dioxirane-mediated epoxidations with caroate (KHSO_5_) as a green oxidant.

This method extends our previous approach on the Merrifield’s resin [17] simplifying the catalyst preparation and widening the substrate scope. Results, coupled with data from chemical characterization of the plasma-treated silica gel, suggest a number of strong points of this protocol: (i) an increased reactivity of ketone (**2**) adsorbed onto the perfluorinated matrix, (ii) an activating effect of the support towards caroate by means of a multiple weak hydrogen-bond network, with generation of singlet oxygen; (iii) the possibility of performing two different kinds of olefin transformations (either epoxidation or double bond cleavage) by means of the simple choice of starting reagents; (iv) the opportunity of extending epoxidation to unsaturated fatty esters like methyl oleate and the triglyceride soybean oil, thus producing bulk chemicals of great practical importance such as ESO; (v) the faculty of recycling the catalyst up to five times and (vi) the high catalyst activity with a total TON value of 206 ca.

Finally, the generation of singlet oxygen also suggests the possibility of using these new perfluorinated silica gel matrixes for applications in photodynamic therapy [39]. All these significant benefits are in accordance with the rules of green chemistry and can provide to the method flexibility and a good potential for industrial applications [40].

## Figures and Tables

**Table 1:**
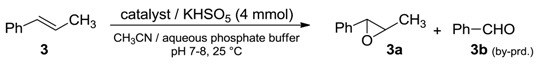
Epoxidation of alkene 3 with potassium caroate ^a^.

Run	Catalyst	Loading ^b^ (%)	Time (h)	Conv. ^c^ (%)	Selectivity (%) ^c^		Leaching
					3a	3b	
1	**2**	9.7	5	90	96	--	--
2	**2**-(**A**)	9.7	4	75	72	28	yes
3	**2**-(**B**)	9.7	4	95	68	29	yes
4	**2**-(**C**)	9.7	4	97	83	17	none
5	**2**-(**C**)	14.6	2	95	86	14	none
6	**2**-(**C**)	9.7	2	80	84	16	none
7	**2**-(**C**)	4.8	2	90	68	32	none
8	**2**-(**C**)	0.0	2	80	-	93	none
9 ^d^	**2**-(**C**)	4.8	4	95	95	5	none

^a^ *Reaction conditions*: alkene (**3**) (1 mmol), heterogeneous catalyst (100 mg, with loaded ketone (**2**) as indicated), caroate (4 mmol, corresponding to 2.86 mmol/g of Caroat^®^ triple salt, iodometry), CH_3_CN/aq. buffer, T = 25 °C. ^b^ Mol% of dioxirane precursor **2** respect to substrate **3**. ^c^ Evaluated by GLC. ^d^ Oxidant/substrate ratio of 6:1 was used in the presence of NaN_3_ (30 mg, 0.46 mmol) as an additive.

**Table 2:** XPS Atomic composition of supports and catalyst **2**-(**C**) ^a^.

Run	Support	C%	O%	Si%	F%
1	C_8_-silica gel	27 ± 2	49 ± 1	24 ± 1	/
2	Perfluorin. silica gel 60 (A)	17 ± 3	42 ± 2	24 ± 2	17 ± 3
3	Perfluorin. silica gel grafted (B)	19 ± 2	35 ± 3	22 ± 3	24 ± 3
4	Perfluorin. silica gel coated (C)	41 ± 3	2 ± 1	1 ± 1	56 ± 3
5	Catalyst 2-(C)	32 ± 3	26 ± 3	7 ± 2	35 ± 3

^a^ Maximum relative standard deviation 3% ca.

**Table 3:**
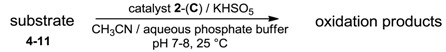
Substrate scope in the alkene epoxidation promoted by catalyst **2**-(**C**) ^a^.

Run	Substrate	Oxd./Sub. ^b^ Ratio	Time (h)	Conv. ^c^ (%)	Products (Yield %) ^d^
1	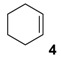	4	2	98	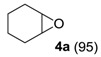
2	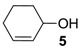	4	1	98	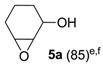
3	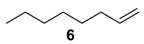	8	12	15	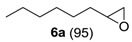
4	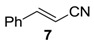	4	48	16	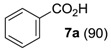
5	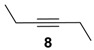	8	4	98	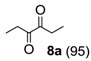
6	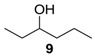	12	48	2	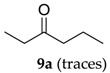
7	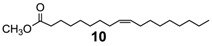	4	4	95	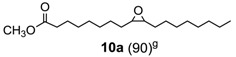
8	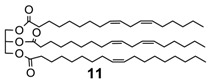	10	9	95	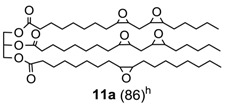

^a^ *Reaction conditions*: substrate (1 mmol), 100 mg of catalyst **2**-(**C**) (bearing 9.7 × 10^−2^ mmol of **2** per 100 mg), caroate as indicated (Caroat^®^ triple salt, iodometry), CH_3_CN/aq. buffer, T = 25 °C. ^b^ KHSO_5_ to substrate molar ratio. ^c^ Evaluated by GLC. ^d^ Isolated yields. ^e^ Mixture of syn/anti stereoisomers in a 60:40 ratio ca., as determined by GC-MS. ^f^ A 15% of cyclohexanone was detected by GC-MS. ^g^ GLC yield. ^h^ Evaluated by NMR (Figure S3 in Supporting Information)

## Supplementary Materials

The following are available online, Table S1: XPS characterization of Catalyst **2-**(**C**); Figure S1: XPS C1s region, curve fitting for **2-**(**C**); Figure S2: XPS O1s region, curve fitting for catalyst **2-**(**C**); Figure S3: ^1^H-NMR spectrum of ESO.
