# Supplementary material for: Heterogenization of Ketone Catalyst for Epoxidation by Low Pressure Plasma Fluorination of Silica Gel Supports
Source: Molecules. 2017 Nov 30;22(12):2099. doi: 10.3390/molecules22122099 (PMC6149926; doi:10.3390/molecules22122099)
Supplement: Supplementary file 1 [file molecules-22-02099-s001.pdf]

Supplementary material

Heterogenization of ketone catalyst for epoxidation by low pressure plasma fluorination of silica gel supports

Lucia D'Accolti,[1,2] * Nicoletta De Vietro,[1] Caterina Fusco,[2] Angelo Nacci,[1,2] Francesco Fracassi[1,3]*

[1] Dipartimento di Chimica, Università di Bari "A. Moro", Via Orabona 4, 70126 Bari, Italy
[2] ICCOM-CNR, SS Bari, Via Orabona 4, 70126 Bari, Italy;
[3] NANOTEC-CNR, c/o Department of Chemistry, University of Bari "Aldo Moro", via Orabona 4, Bari, Italy

* Correspondence: lucia.daccolti@uniba.it, Tel.: +39 080 5442068; francesco.fracassi@uniba.it, Tel.: +39 080 5442009

Academic Editor: name
Received: date; Accepted: date; Published: date

corresponding author: lucia.daccolti@uniba.it, francesco.fracassi@uniba.it

(8 pages, including this cover)

Contents:

1. *Table S1* XPS characterization of Catalyst **2-C** *p. 2*
2. *Figure S1* XPS C1s region, curve fitting for (**2-C**) *p. 3*
3. *Figure S2* XPS O1s region, curve fitting for catalyst (**2-C**) *p. 4*
4. *Figure S3* ^{1}H-NMR spectrum of ESO *p. 5*

Table S1: Components used for the curve-fitting of the high-resolution C1s and O1s XPS signals of Catalyst **2-C.**

**Signal[a]**	**component**	**BE (eV)**	**assignment**
C1s	C1	285.0 ±0.2	C-C/C-H
	C2	286.5 ±0.3	**C-C=O/**C-O
	C3	287.7 ±0.3	C-CF/C-O-C=O
	C4	289.4 ±0.2	CF/C=O/O-C-O/ O-**C**=O
	C5	291.5 ±0.3	CF_2
	C6	293.7 ±0.2	CF_3
O1s	O1	532.8 ±0.3	**O=C**/O-CH_x
	O2	534.2 ±0.3	O-CF_x

[a] G.Beamson, D. Briggs, High Resolution XPS of Organic Polymers, the scienta ESCA 300 data-base, J. Wiley & Sons, Chichester, (1992).

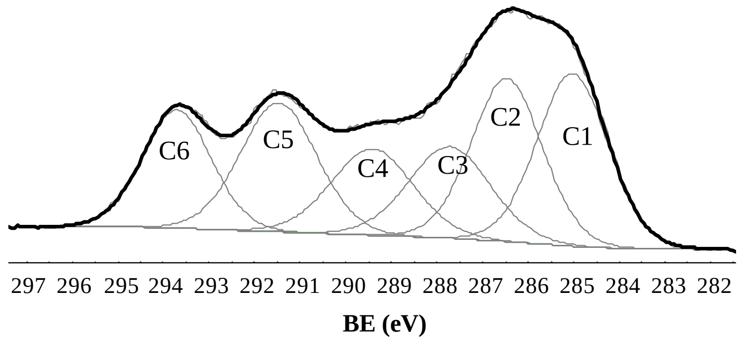

peak	area %
C1	21.7±0.3
C2	20.5±0.3
C3	13.3±0.2
C4	12.6±0.2
C5	17.5±0.3
C6	14.4±0.3

Figure S1. XPS curve fitting for catalyst (**2-C**) C1s region

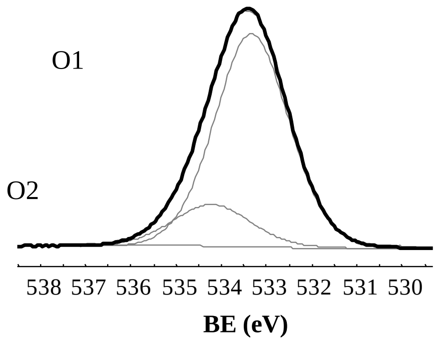

peak	area %
O1	82.6±0.3
O2	17.4±0.3

Figure S2. XPS curve fitting for catalyst (**2-C**) O1s region

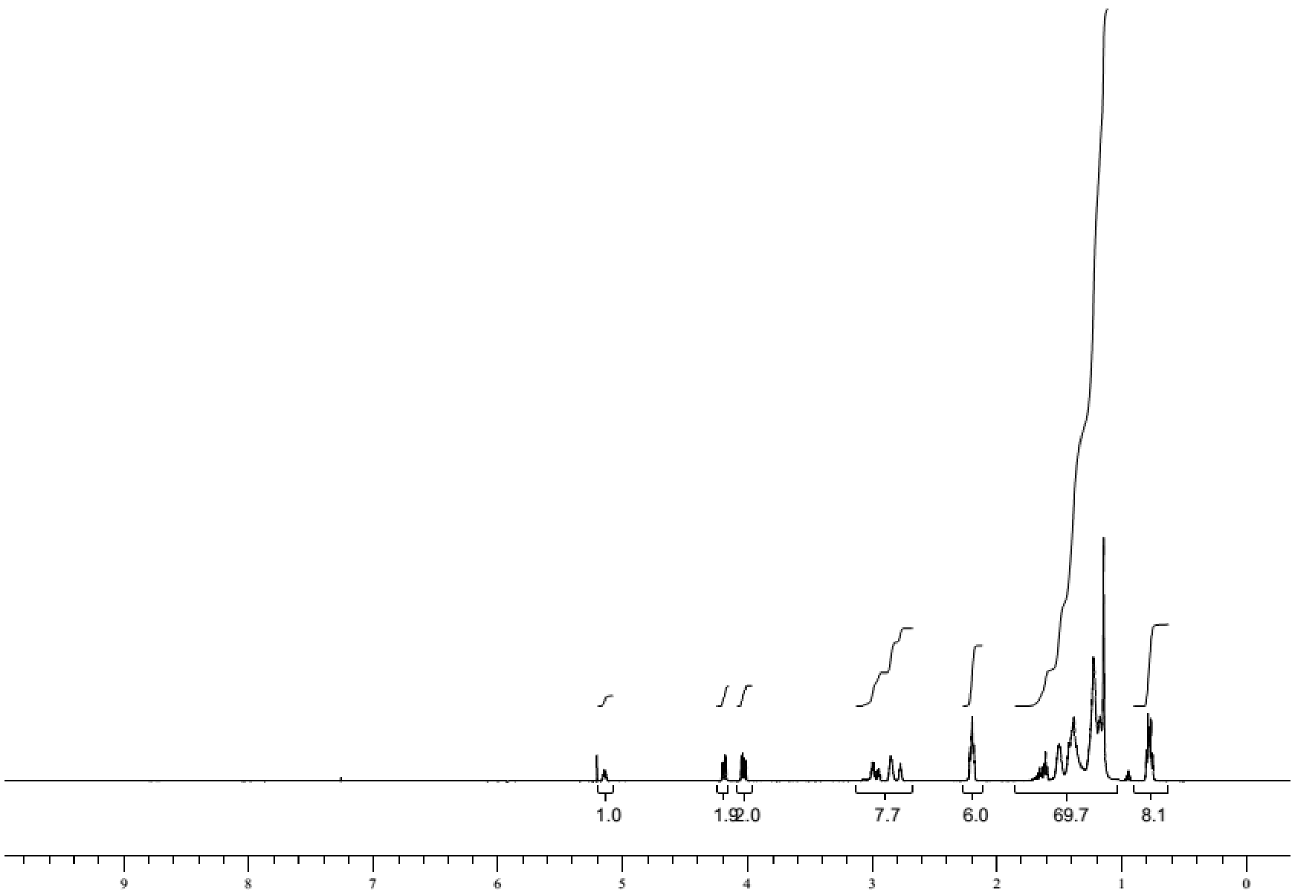

Figure S3. ^{1}H NMR ($CDCl_3$, 500 MHz) spectrum of ESO
